# Tubedown regulation of retinal endothelial permeability signaling pathways

**DOI:** 10.1242/bio.010496

**Published:** 2015-07-03

**Authors:** Nhu Ho, Robert L. Gendron, Kindra Grozinger, Maria A. Whelan, Emily Anne Hicks, Bimal Tennakoon, Danielle Gardiner, William V. Good, Hélène Paradis

**Affiliations:** 1 Division of BioMedical Sciences, Department of Medicine, Memorial University of Newfoundland, St. John's, Newfoundland and Labrador, CanadaA1B 3V6; 2 Smith-Kettlewell Eye Research Institute, San Francisco, CA 94115, USA

**Keywords:** Endothelium, Permeability, Retina, Signaling, Tubedown, c-Src, Cortactin

## Abstract

Tubedown (Tbdn; Naa15), a subunit of the N-terminal acetyltransferase NatA, complexes with the c-Src substrate Cortactin and supports adult retinal homeostasis through regulation of vascular permeability. Here we investigate the role of Tbdn expression on signaling components of retinal endothelial permeability to understand how Tbdn regulates the vasculature and supports retinal homeostasis. Tbdn knockdown-induced hyperpermeability to Albumin in retinal endothelial cells was associated with an increase in the levels of activation of the Src family kinases (SFK) c-Src, Fyn and Lyn and phospho-Cortactin (Tyr421). The knockdown of Cortactin expression reduced Tbdn knockdown-induced permeability to Albumin and the levels of activated SFK. Inhibition of SFK in retinal endothelial cells decreased Tbdn knockdown-induced permeability to Albumin and phospho-Cortactin (Tyr421) levels. Retinal lesions of endothelial-specific Tbdn knockdown mice, with tissue thickening, fibrovascular growth, and hyperpermeable vessels displayed an increase in the levels of activated c-Src. Moreover, the retinal lesions of patients with proliferative diabetic retinopathy (PDR) associated with a loss of Tbdn expression and hyperpermeability to Albumin displayed increased levels of activated SFK in retinal blood vessels. Taken together, these results implicate Tbdn as an important regulator of retinal endothelial permeability and homeostasis by modulating a signaling pathway involving c-Src and Cortactin.

## INTRODUCTION

Endothelial cell permeability regulates interstitial tissue fluid balance and the transport of a range of molecules across the vessel wall which are important for the maintenance of tissue homeostasis (reviewed in Mehta and Malik, 2006). The transport of plasma proteins and solutes across the endothelium occurs via two different routes. The transcellular route is mediated by an intracellular vesicular transport while the paracellular route occurs through interendothelial junctions (Komarova and Malik, 2010). Endothelial hyperpermeability is a pathobiological complication shared by a number of different diseases (reviewed in Nagy et al., 2012). Increased retinal vascular permeability is one of the early pathophysiological mechanisms underlying retinal neovascular diseases such as PDR and the wet form of age-related macular degeneration (AMD) (Leto et al., 2001; Kumar et al., 2009; Giani et al., 2011; Lutty, 2013). A role for extravasation of proangiogenic and proinflammatory factors during the breakdown of the endothelial barrier in neovascular retinopathies is now well established (Friedlander et al., 1996; Ljubimov et al., 1996; Paques et al., 1997; Campochiaro and Hackett, 2003; Bhutto and Lutty, 2012) and represents a clinically important aspect of the disease (Giani et al., 2011; Antonetti et al., 2012). A better understanding of the molecular and cellular mechanisms underlying retinal endothelial barrier maintenance and the events associated with retinal barrier loss are thus essential to develop new strategies to prevent or treat neovascular retinopathies.

Tubedown (Tbdn, also referred to as Narg1, mNat1, NATH, Naa15) has been defined from previous research in our laboratories as a regulator of angiogenesis and vascular permeability in adult retinal blood vessels (Paradis et al., 2002, 2008; Wall et al., 2004; Gendron et al., 2010). Tbdn is one of two homologues of the Nat1 family of N-terminal acetyltransferase subunits that binds to the catalytic subunit Ard1 (also referred to as Naa10) (reviewed in Kalvik and Arnesen, 2013). Tbdn is a developmentally regulated protein expressed at high levels during embryogenesis and in some cancers (Gendron et al., 2000; Sugiura, et al., 2003; Kalvik and Arnesen, 2013). In both yeast and mammals, the Nat1/Ard1 complex (also referred as NatA) has been shown to play an important role in the regulation of a broad range of cellular processes varying from cell growth to cellular differentiation and a variety of potential substrates have been reported (Gendron et al., 2001; Paradis et al., 2002, 2008; Kimura et al., 2003; Sugiura et al., 2003; Geissenhoner et al., 2004; Wall et al., 2004; Wang et al., 2004; Asaumi et al., 2005; Kalvik and Arnesen, 2013; Myklebust et al., 2015). One well established function of the Nat1/Ard1 complex is the N-terminal acetylation of nascent proteins (Kalvik and Arnesen, 2013). Recent structural studies have provided evidence that yeast Nat1 alters the active site of Ard1 mediating sequence specific acetylation patterns of substrates (Liszczak et al., 2013). In mammals during adulthood, high levels of Tbdn are restricted to few tissues including the retinal vasculature (Gendron et al., 2001, 2010; Paradis et al., 2002, 2008). Previous research has shown that Tbdn protein expression is suppressed in eyes from patients with neovascular retinopathies including PDR (Gendron et al., 2001), retinopathy of prematurity (Gendron et al., 2006) and AMD (Gendron et al., 2010). In addition, Tbdn knockdown in retinal endothelial cells *in vitro* and in animal models has been associated with increases in retinal angiogenesis and retinal blood vessel hyperpermeability to Albumin, a hallmark of neovascular retinopathy (Vinores et al., 1989, 1990, 1993; Knudsen et al., 2002; Paradis et al., 2002, 2008; Wall et al., 2004).

Tbdn has also been shown to be part of a complex with the actin binding protein Cortactin (Paradis et al., 2008). Cortactin regulates actin assembly, cytoskeletal remodeling, endothelial barrier integrity, and was originally identified as a major substrate of the tyrosine kinase c-Src (Weed and Parsons, 2001; Daly, 2004; Mehta and Malik, 2006). Cortactin is phosphorylated by c-Src at tyrosine residues 421, 466, and 482 (Daly, 2004). Phosphorylation of Cortactin at Tyr421 by c-Src regulates cytoskeleton remodeling and coordination of membrane dynamics including endocytosis (Cosen-Binker and Kapus, 2006; Ammer and Weed, 2008). Albumin permeability in endothelial cells is mediated by transcytosis and involves activation of c-Src (reviewed in Hu and Minshall, 2009). The binding of Albumin to its cell surface receptor gp60 promotes clustering of the receptor and recruitment of Caveolin-1 to the complex (Komarova and Malik, 2010). This event leads to the recruitment and activation of the G-protein Gαi promoting phosphorylation of c-Src at Tyr416 and activation (Komarova and Malik, 2010). Activated c-Src phosphorylates components of the endothelial permeability pathway such as Caveolin-1, Dynamin-2 and gp60, facilitating caveolar scission, endocytosis and transcellular vesicular transport of Albumin (Kim et al., 2009; Komarova and Malik, 2010). In addition, the phosphorylation of Cortactin and Dynamin by c-Src plays an important role in mediating and stimulating receptor-mediated endocytosis (Cao et al., 2010). The phosphorylation of Cortactin by c-Src enhances Cortactin binding affinity to Dynamin (Zhu et al., 2007) an essential step for vesicle formation at the plasma membrane (Cao et al., 2003). The specific signaling mechanisms by which c-Src facilitates transport of Albumin across endothelial cell from the luminal to the abluminal side of blood vessels is not completely understood.

In this study, we explored the notion that Tbdn regulates intracellular components of the Albumin permeability signaling pathway in retinal endothelial cells *in vitro* and *in vivo*. We demonstrate that Tbdn knockdown leads to the activation of the c-Src/Cortactin pathway both *in vitro* and *in vivo*. C-Src and Cortactin have emerged as important factors in the signaling pathways mediating endothelial cell permeability (Mehta and Malik, 2006; Komarova and Malik, 2010; Schnoor et al., 2011). The work herein provides new insight into the role that Tbdn plays in relation to SFK members and Cortactin signaling in the regulation of retinal endothelial permeability and the maintenance of retinal homeostasis.

## RESULTS

### Tbdn knockdown in retinal endothelial cells leads to up-regulation of activated Src family of kinases pathway

**Fig. 1.**
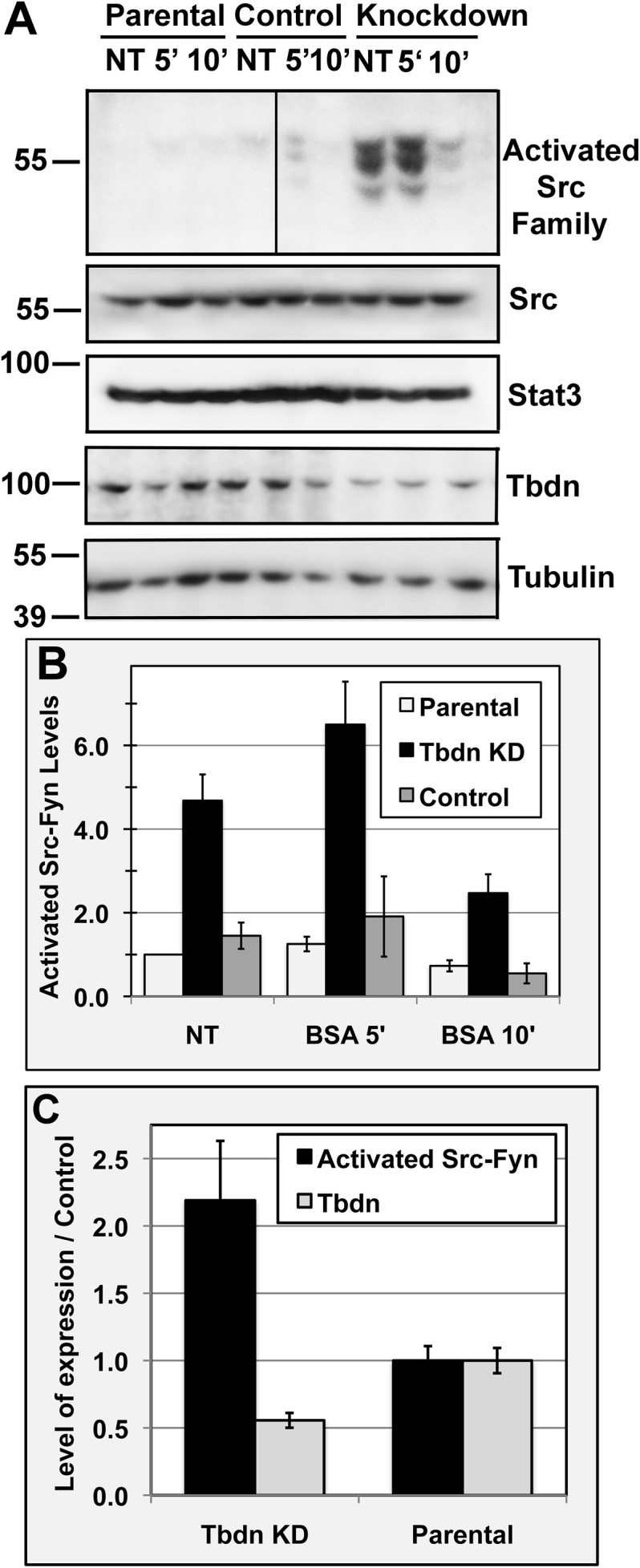
**Tbdn knockdown in retinal endothelial cells leads to up-regulation of activated Src family of kinases.** RF/6A parental (Parental), Tbdn knockdown (Knockdown) and control (Control) retinal endothelial cell clones were serum starved followed by no treatment (NT) or stimulation with serum Albumin (BSA) for 5 (5′) and 10 (10′) min. (A) Samples were analyzed by western blotting for levels of activated SFK (Activated Src Family), total c-Src (Src), and Stat3 for loading control or Tbdn and Tubulin for loading control. A representative experiment is shown. Results showed a higher constitutive level of activation of SFK (identified as c-Src co-migrating with Fyn (top band, relative molecular weight 60 kDa) and Lyn (middle and lower bands, relative molecular weight 56 kDa and 53 kDa; see Fig. 5A) in Tbdn knockdown clones which express reduced levels of Tbdn compared to the parental cells and control clones. For the top panel three skipped lanes between the Control NT lane and Control 5′ lane were cropped in the image. (B) Average quantitation of western blot analyses of activated c-Src-Fyn (60 kDa band in A) over loading control in RF/6A parental cells (Parental), two Tbdn knockdown clones (Tbdn KD) and two control clones (Control) for which representatives are shown in A. Results are expressed as fold relative to non-treated (NT) parental cells. Similar results were obtained for activated c-Src-Fyn over total c-Src or total Fyn (supplementary material Fig. S1). (C) Average quantitation of western blot analyses of activated c-Src-Fyn (60 kDa band in A) and Tbdn over respective loading control in RF/6A parental cells (Parental) versus Tbdn knockdown clones (Tbdn KD) grown in the presence of serum (*P*<0.034). B and C, means±s.e.m. of six experiments are indicated.

Previous studies have shown that Tbdn regulates retinal endothelial permeability to Albumin (Paradis et al., 2008; Gendron et al., 2010). In this study we assessed the relationship between Tbdn and known regulators of vascular endothelial permeability in the retina. The activation of the tyrosine kinase c-Src plays key roles in the regulation of microvascular barrier function and various endothelial responses including permeability to Albumin (Kim et al., 2009; Hu and Minshall, 2009). Moreover, Tbdn was previously found in a complex with Cortactin, a filamentous-actin binding protein and prominent substrate of c-Src (Weed and Parsons, 2001; Paradis et al., 2008). The effect of Tbdn expression on major components of the Albumin permeability pathway, c-Src and Cortactin, were first investigated *in vitro* in the retinal endothelial cell line RF/6A. RF/6A cell clones stably knocked down for Tbdn by expression of an antisense *Tbdn* cDNA fragment that exhibit increased transcellular permeability to Albumin (Paradis et al., 2002, 2008) and RF/6A cells transiently knocked down for Tbdn expression using siRNA were both used to examine the effect of Tbdn expression on components of the Albumin permeability pathway. The effects of Tbdn knockdown on the levels of activated SFK were studied by western blot using a phospho-Src family (Tyr416) antibody. Three bands with relative molecular mass of 60 kDa, 56 kDa and 53 kDa were detected by western blot analysis of Tbdn knockdown RF/6A retinal endothelial cell clones using the phospho-Src family (Tyr416) antibody (Fig. 1A). Immunoprecipitations with antibodies directed against individual Src family members c-Src, Fyn and Lyn followed by western blot with phospho-Src family (Tyr416) antibody confirmed the identity of the bands recognized by the activated Src family antibody as c-Src, Fyn, and Lyn in Tbdn knockdown RF/6A cells (not shown and see Fig. 5A). Activated phospho-c-Src (Tyr416) and activated phospho-Fyn co-migrated on SDS-PAGE at a relative molecular weight of approximately 60 kDa while activated phospho-Lyn corresponded to the two molecular weight bands of approximately 53 kDa and 56 kDa. These observations are consistent with previously reported molecular weights of c-Src, Fyn and Lyn (Lannutti et al., 2006; Zheng et al., 2008). Respective immunoprecipitations of the three kinases showed that the levels of activated Fyn in RF/6A cells knocked down for Tbdn was minimal compared to the levels of activated c-Src and activated Lyn (not shown and see Fig. 5A).

**Fig. 2.**
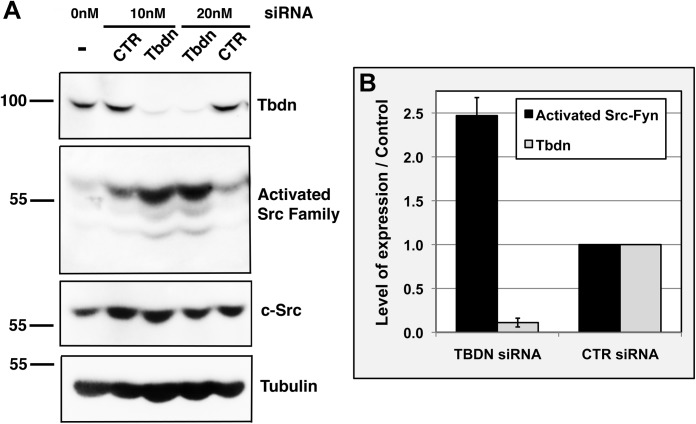
**Tbdn knockdown by siRNA in retinal endothelial cells leads to up-regulation of activated Src family of kinases.** (A) Tbdn expression was knocked down by transfection of *Tbdn* siRNA (10 and 20 nM *Tbdn* siRNA) compared to control siRNA (10 and 20 nM CTR siRNA) or no siRNA in RF/6A retinal endothelial cells. Samples were analyzed by western blotting for levels of Tbdn, activated SFK (Activated Src Family), total c-Src (Src), and Tubulin for loading control. A representative experiment is shown. (B) Quantitation of western blot analyses of Tbdn and activated c-Src-Fyn (top band shown in A, relative molecular weight 60 kDa) over loading control (Tubulin for Tbdn and total c-Src for activated c-Src-Fyn) for which representatives are shown in A. Results are expressed as fold relative to control cells treated with control siRNA (*P*≤0.033). Similar results were obtained for activated c-Src-Fyn over Tubulin. Means±s.e.m. of 5 experiments are indicated.

Levels of activated SFK relative to loading control or total c-Src or total Fyn were up-regulated in two different Tbdn knockdown RF/6A retinal endothelial cell clones compared to the parental cells and two different control cell clones (Fig. 1 and supplementary material Fig. S1; Paradis et al., 2002, 2008). These same RF/6A cell clones showing up-regulation of activated SFK levels were previously reported to exhibit increased transcellular permeability to Albumin compared to controls (Paradis et al., 2008). Moreover, similar results were obtained when Tbdn was knocked down by siRNA (Fig. 2). In serum starved cells, constitutive knockdown of Tbdn expression by stable transfection of an antisense *Tbdn* cDNA fragment resulted in 5- to 3-fold increase (*P*<0.003) in the levels of activated c-Src-Fyn with a relative molecular mass of 60 kDa compared to parental cells and control clones (Fig. 1A and B). Under normal growth conditions in the presence of 10% fetal bovine serum (FBS), Tbdn knockdown clones exhibited a 2-fold increase in the levels of activated c-Src-Fyn compared to parental cells (*P*<0.034; Fig. 1C). Similarly, in the presence of 10% FBS, transient Tbdn knockdown using siRNA resulted in an approximately 2.5-fold increase in activated c-Src-Fyn compared to control siRNA (*P*≤0.033; Fig. 2B).

To evaluate the effect of Tbdn knockdown on the activation of the Albumin permeability pathway, serum deprived RF/6A retinal endothelial cells stably knocked down for Tbdn or control cells were stimulated with bovine serum Albumin (BSA) for 5 and 10 min and levels of activated SFK analyzed. Albumin stimulation of Tbdn knockdown, parental and control clones resulted in a transient increase in the levels of activated SFK, with the highest levels observed in the Tbdn knockdown clones at 5 min (*P*<0.015; Fig. 1A,B). Western blots analyses showed no significant changes in the levels of total c-Src or total Fyn relative to the loading control between the different clones (Fig. 1A and supplementary material Fig. S1).

**Fig. 3.**
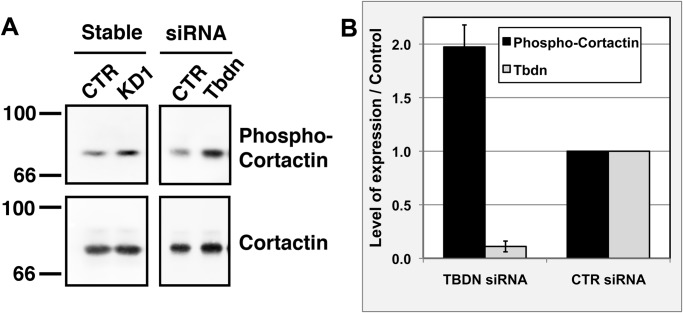
**Tbdn knockdown in retinal endothelial cells leads to an increase in phospho-Cortactin (Tyr421).** (A) Tbdn expression was knocked down in RF/6A retinal endothelial cells by either stable transfection of an antisense *Tbdn* construct (Stable KD1) or transient transfection of a *Tbdn* siRNA (siRNA Tbdn). Cell extracts from Tbdn knockdowns and from respective controls (Stable CTR or siRNA CTR) were analyzed for levels of phospho-Cortactin (Tyr421) versus total Cortactin by western blotting. Representative results are shown. (B) Levels of phospho-Cortactin (Tyr421) over total Cortactin and levels of Tbdn over loading control were quantified in controls (CTR siRNA) or Tbdn knockdown (Tbdn siRNA) (*P*<0.018). Data is expressed as mean±s.e.m. of 4 experiments.

To further study the mechanism by which Tbdn regulates the Albumin transcellular pathway, we examined the effect of Tbdn knockdown on the levels of phospho-Cortactin (Tyr421) which was previously identified as a c-Src target (Daly, 2004). RF/6A retinal endothelial cells were knocked down for Tbdn expression as above either transiently by siRNA or stably by transfection with an antisense *Tbdn* cDNA fragment (Figs 1 and 2; Paradis et al., 2002, 2008). Tbdn knockdown by either stable transfection or transient transfection was associated with an increase in phospho-Cortactin (Tyr421) levels whereas the levels of total Cortactin did not vary (Fig. 3A). Tbdn knockdown resulted in a 2-fold increase in phospho-Cortactin (Tyr421) over the levels of total Cortactin (*P*<0.018; Fig. 3B).

### Cortactin regulates Albumin permeability and Src family of kinases activation in retinal endothelial cells knocked down for Tbdn

**Fig. 4.**
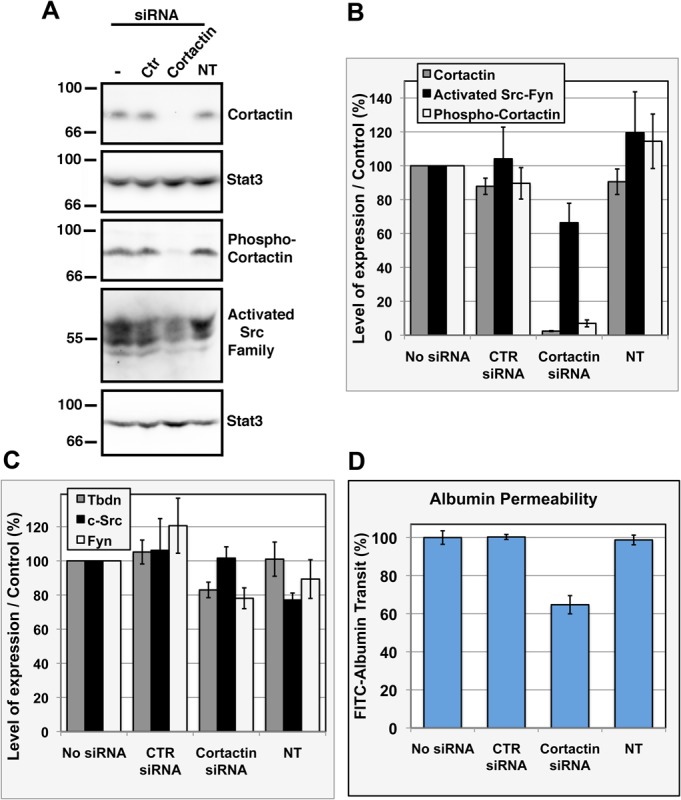
**Role of Cortactin in the regulation of Src family of kinases and Albumin permeability in retinal endothelial cells knocked down for Tbdn.** Cortactin expression was knocked down by transfection of *Cortactin* siRNA (Cortactin siRNA) compared to control siRNA (CTR siRNA), no siRNA (No siRNA) and non-transfected (NT) RF/6A retinal endothelial cells knockdown for Tbdn. Samples were analyzed by western blotting for levels of Cortactin, activated SFK (Activated Src Family), phospho-Cortactin (Tyr421), total c-Src (Src), total Fyn, Tbdn, and Stat3 or Tubulin for loading control; and Albumin permeability assay. (A) Representative western blot analysis is shown. (B,C) Quantitation of western blot analyses of indicated protein over loading control (Tubulin or Stat3). Relative to cells transfected with control siRNA (CTR siRNA) or no siRNA (No siRNA) and non-transfected (NT) cells, *Cortactin* siRNA transfected cells exhibited reduced levels of Cortactin (*P*<0.0001), reduced levels of activated Src-Fyn (*P*≤0.049), and reduced levels of phospho-Cortactin (*P*<0.009) (B), while Tbdn, total c-Src and total Fyn were not significantly changed (C). (D) The transit of FITC-Albumin across *Cortactin* siRNA transfected retinal endothelial cells knockdown for Tbdn (Cortactin siRNA) was reduced compared to controls (CTR siRNA, No siRNA and NT) (*P*<0.0001). B-D: Results are expressed as percentage relative to control cells transfected without siRNA and represent the means±s.e.m. of at least 3 experiments.

Since Tbdn forms a complex with Cortactin (Paradis et al., 2008), we used a *Cortactin* siRNA (Schnoor et al., 2011) to further explore the importance of Cortactin on Albumin permeability and Src family of kinases activation in the context of suppressed Tbdn expression in retinal endothelial cells (Fig. 4). Cortactin knockdown (*P*<0.0001) in retinal endothelial cells suppressed for Tbdn expression was associated with a decrease in both the levels of activated c-Src-Fyn (*P*<0.05) and Albumin permeability (*P*<0.0001) compared to cells transfected without siRNA, cells transfected with control siRNA and non-transfected cells (Fig. 4A,B,D). The levels of phospho-Cortactin (Tyr421) (Fig. 4A,B) were significantly downregulated in cells transfected with *Cortactin* siRNA compared to controls (*P*<0.009). The knockdown of Cortactin expression had no significant effect on the levels of Tbdn (*P*>0.38), total c-Src (*P*>0.33), Fyn (*P*>0.21) or cell growth (*P*>0.99) compared to controls (Fig. 4C and not shown).

### Src family of kinases inhibition in retinal endothelial cells knocked down for Tbdn leads to decreased Albumin permeability

**Fig. 5.**
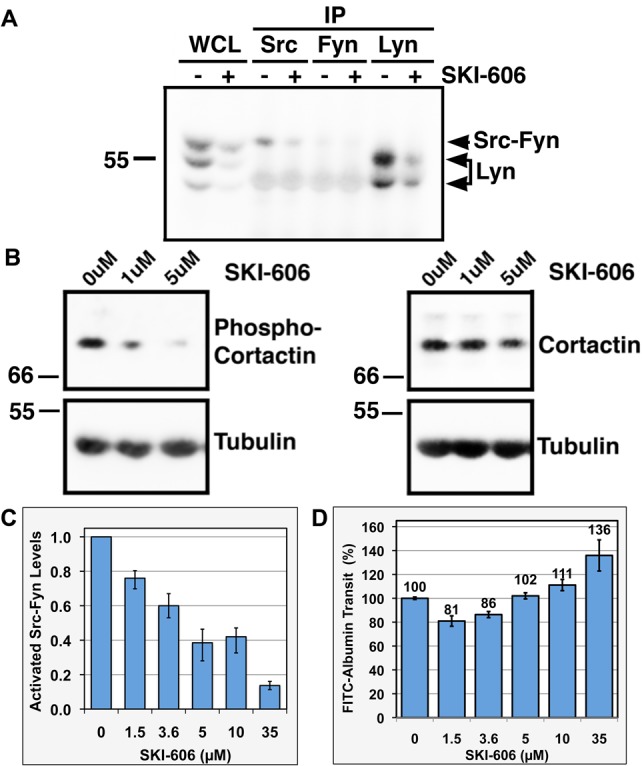
**Effect of Src family of kinases inhibitor SKI-606 on the transcellular permeability of retinal endothelial cells knocked down for Tbdn.** (A) RF/6A retinal endothelial cells stably knocked down for Tbdn were treated with 1.5 μM of SKI-606 or vehicle for 24 h. Whole cell lysates (WCL) and c-Src, Fyn and Lyn immunoprecipitations (IP) were analyzed by western blots with a phospho-Src family (Tyr416) antibody (shown) or with either c-Src, Fyn or Lyn antibodies to confirm efficacy of immunoprecipitations (not shown). Activated SFK western blot of c-Src and Fyn immunoprecipitations revealed a non-specific band at 54 kDa. Representative results of 4 experiments are shown. (B) Retinal endothelial cells stably knocked down for Tbdn were treated with indicated amount of SKI-606 or vehicle for 20 h. Whole cell lysates were analyzed by western blotting for phospho-Cortactin (Tyr421), Cortactin and Tubulin as loading controls. Representative results of at least 3 experiments are shown. (C) Relative levels of activated c-Src-Fyn (represented in panel A WCL lane, top band 60 kDa) quantified by western blot analysis for phospho-Src family (Tyr416) of RF/6A retinal endothelial cells stably knocked down for Tbdn treated with indicated amount of SKI-606 under high cell density conditions (optimal permeability assay conditions). Data is expressed as mean±s.e.m. of 4 experiments. (D) Percentages of FITC-Albumin transit across a monolayer of retinal endothelial cells stably knocked down for Tbdn treated with various concentrations of SKI-606 as indicated or with vehicle only (0 µM). Vehicle treated cells are significantly different from all SKI-606 treated cells (*P*<0.02) except 5 µM SKI-606. Data is expressed as mean±s.e.m. of 6 experiments.

The role of c-Src in Tbdn regulation of retinal endothelial cell permeability, was examined using the Src family of kinases inhibitor SKI-606 (Golas et al., 2003) and *c-Src* siRNA (Zheng et al., 2008). In subconfluent cell monolayers, 1.5 µM of SKI-606 was sufficient to achieve significant reduction in the level of activated SFK (Fig. 5A). Under these conditions, SKI-606 effectively inhibited c-Src and Lyn but Fyn was more modestly inhibited in retinal endothelial cells knocked down for Tbdn (Fig. 5A). Reduction in the levels of activated SFK correlated with a decrease in phospho-Cortactin (Tyr421), while expression levels of both Cortactin or c-Src (not shown) in the SKI-606 treated versus vehicle treated cells were not significantly different (Fig. 5B). However, as previously reported in other cell types (Golas et al., 2003; Coluccia et al., 2006; Elliott et al., 2011), high concentrations of SKI-606 were associated with reduced cell survival. Similarly, a ∼40% or more c-Src knock down by 2.5 nM and above of siRNA resulted in a decrease in phospho-Cortactin (Tyr421) levels and was associated with reduction in cell survival as previously reported for other cells types (Coluccia et al., 2006; Zheng et al., 2008; supplementary material Fig. S2 and data not shown).

Despite the impact of SKI-606 and c-Src knockdown on the survival of the retinal endothelial cells, the effect of SKI-606 and *c-Src* siRNA (1 nM to 5 nM) on the Albumin permeability of RF/6A retinal endothelial cells knocked down for Tbdn was next examined (Fig. 5D and supplementary material Fig. S2). As previously published (Paradis et al., 2008), RF/6A cells knocked down for Tbdn consistently exhibited a higher rate of FITC-Albumin transit across a confluent cell monolayer compared to control cell clones or non-transfected cells (∼25%; supplementary material Fig. S2B). For confluent cell monolayers, higher levels of SKI-606 were required to achieve a similar reduction in the levels of activated SFK than in subconfluent monolayers. Western blot analyses of lysates from confluent monolayers of RF/6A retinal endothelial cells knocked down for Tbdn and treated with 1.5 µM to 35 µM SKI-606 showed a dose-response decrease of the levels of activated c-Src-Fyn with a relative molecular mass of 60 kDa (21 to 86%) compared to vehicle treated cells (Fig. 5C).

Transcellular permeability assays performed on confluent monolayers of Tbdn knockdown RF/6A cells pre-treated with increasing concentrations of SKI-606 revealed a decrease of approximately 20±4% and 15±3% in the rate of FITC-Albumin transit across the cell monolayer at 1.5 µM and 3.6 µM of inhibitor, respectively (Fig. 5D). However, at concentrations of 5 µM and above of SKI-606, the permeability of the endothelial cells knocked down for Tbdn no longer appeared to be inhibited by the Src family of kinases inhibitor (*P*=0.102). Moreover, at high concentrations of inhibitor (10 µM and above), the rate of FITC-Albumin transit across the cell monolayer was significantly higher than the vehicle treated control (*P*<0.02) and was independent of time. These results suggested that the cell monolayers had become leaky to FITC-Albumin at these higher concentrations of inhibitor. Similarly to the inhibition of c-Src by high dose of SKI-606 treatment, assays for Albumin permeability also suggested that the cell monolayers had become leaky to FITC-Albumin following transfection of 5 nM of *c-Src* siRNA compared to controls (supplementary material Fig. S2B,C).

### Tbdn knockdown in mouse blood vessels leads to increased levels of activated c-Src in the retina

**Fig. 6.**
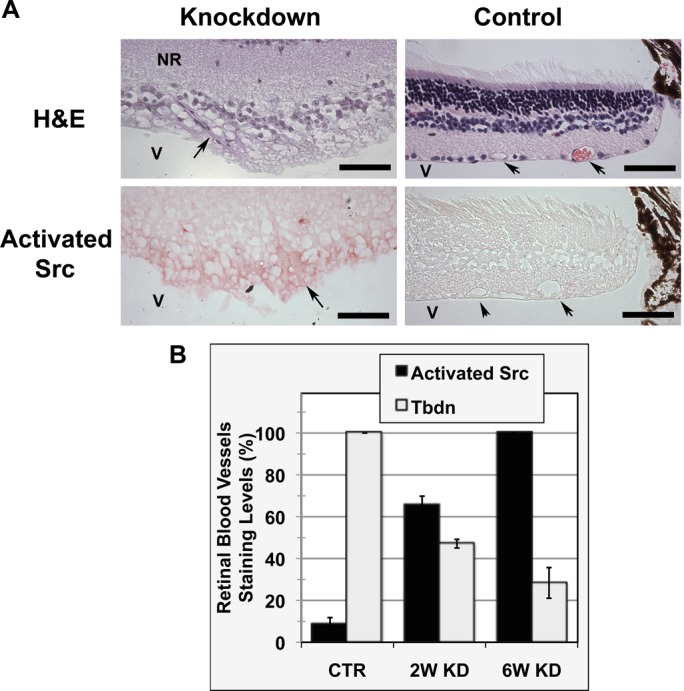
**Levels of activated Src family of kinases in retinal lesions of Tbdn knockdown mice.** (A) Top panels, hematoxylin/eosin (H&E) staining of eye sections from a 6 week-induced endothelial specific Tbdn knockdown mouse retinal lesion showing abnormal blood vessels and retinal thickening compared to a control age-match mouse retina section. Lower panels, immunohistochemical analysis of the levels of activated SFK (shown by red staining) of specimens shown in top panel. Retinal sections stained with no primary antibody showed no staining (not shown). All images are oriented with the vitreous cavity (v) of the eye at the bottom of the panels, blood vessels arrowed, NR: neural retina. 400×, scale bar=50 µm. Representative experiment is shown. (B) Quantitation of levels of Tbdn and activated SFK in retinal blood vessels of lesions from 2-week (2W) and 6-week (6W) induced Tbdn knockdown (KD) compared to normal retinal blood vessels of age-matched control (CTR). Data shown in B is expressed as mean±s.e.m. of at least 3 duplicate experiments in each group.

To determine if Tbdn expression regulates SFK in the retinal vasculature *in vivo*, the levels of activated SFK in a conditional endothelial specific Tbdn knockdown mouse model (Wall et al., 2004) were next examined. This endothelial specific Tbdn knockdown model was previously shown to display retinal lesions characterized by significant thickening of all the retinal layers, abnormal vasculature with fibrovascular growth, and vascular hyperpermeability to Albumin (Wall et al., 2004; Paradis et al., 2008). In comparison to control age-matched mice, increased immunostaining for activated SFK was observed in retinal lesions resulting from endothelial specific Tbdn knockdown (Fig. 6). Quantitation of the immunostaining in retinal blood vessels revealed a 10-fold increase in activated SFK levels in sections of mouse retinal tissues in which Tbdn endothelial expression was knocked down for 6-week compared to control (*P*<0.00001; Fig. 6B). A higher level of expression of activated SFK was observed in retinal neovascular lesions of mice in which Tbdn endothelial expression was knocked down for 6 weeks compared to 2 weeks (*P*<0.0003). This further increase in activated SFK correlated with a further reduction in the levels of Tbdn expression in the retinal neovascular lesions (Fig. 6B).

**Fig. 7.**
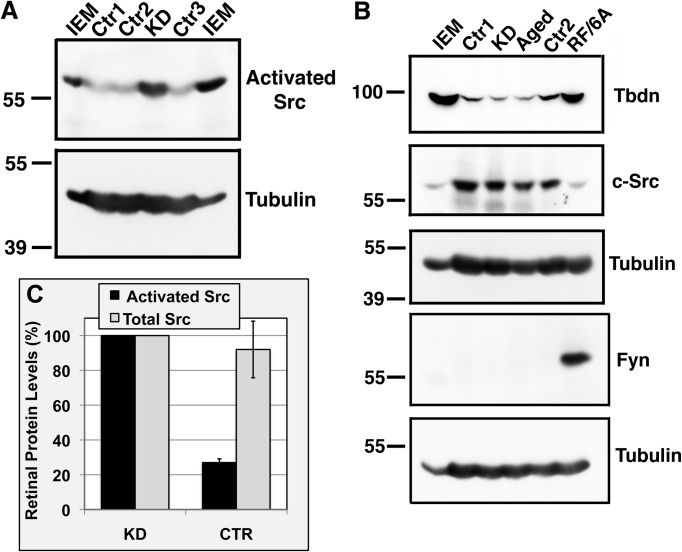
**Increased activated c-Src levels in retina of endothelial specific Tbdn knockdown mice.** (A,B) Representative western blots of retinal lysates from controls (CTR1, CTR2, CTR3), 6-week induced Tbdn knockdown (KD) and aged mice (Aged) were performed using the antibodies indicated. Note Tbdn suppression during aging (Gendron et al., 2010). Cell lysates from IEM mouse embryonic endothelial cells (IEM) and RF/6A retinal endothelial cells (RF/6A) were used as positive controls. Tubulin was used as loading control and for sample integrity. (C) Quantitative analyses of retinal lysate western blots for activated c-Src and total c-Src for which representatives are shown in two previous panels. Levels of activated c-Src are expressed as percent of the maximal levels observed in 6-week induced Tbdn knockdown mice. Means±s.e.m. of 3 experiments are indicated.

To confirm our immunohistochemical data indicating that blood vessels of retinal lesions of endothelial Tbdn knockdown mice are associated with an up-regulation of levels of activated SFK, western blot analyses with phospho-Src family (Tyr416) antibody were performed on isolated mouse retinal tissues in which Tbdn endothelial expression was knocked down for 6-weeks versus control. As shown in Fig. 7, retinal tissues from endothelial Tbdn knockdown mice showed a significant increase (2.5-fold) in the 60 kDa activated Src family protein band (*P*<0.0019) while total levels of c-Src were not significantly changed (Fig. 7). Western blot analyses with phospho-Src family (Tyr416) antibody only revealed one band at approximately 60 kDa in mouse retina (Fig. 7B) instead of 3 bands previously observed in the retinal endothelial cell line RF/6A knocked down for Tbdn (Figs 1A and 5A). These results suggested that activated Lyn is not detectable by western blot in mouse retinal tissues. Moreover, western blot analysis using an antibody against Fyn revealed no detectable expression of Fyn in mouse retinal tissues as compared to RF/6A cells (Fig. 7B). Immunoprecipitation of c-Src followed by western blot with the phospho-Src family (Tyr416) antibody confirmed that the 60 kDa band detected in the mouse retina extract corresponded to c-Src (not shown).

### Human neovascular retinopathy specimens exhibit increased levels of activated Src family of kinases

**Fig. 8.**
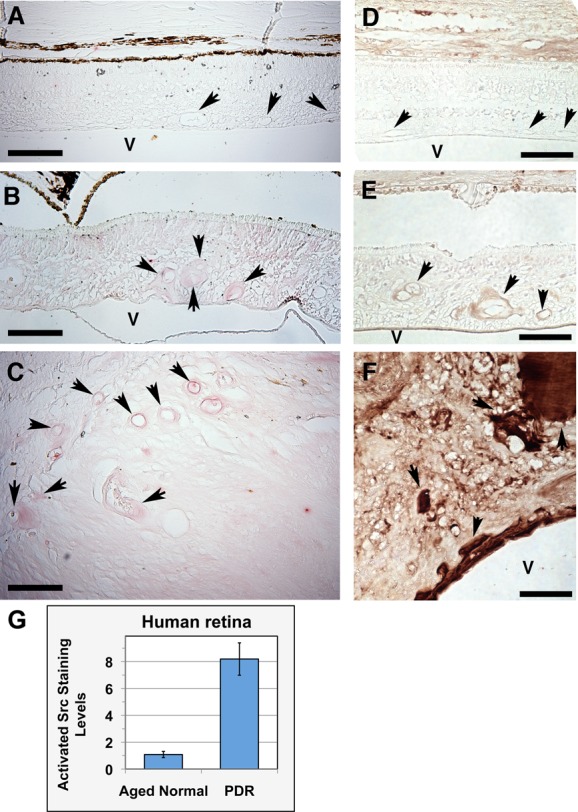
**Levels of activated** **Src family of kinases in retinal lesions of human neovascular retinopathy.** (A-C) Immunohistochemical analysis of the levels of activated SFK (shown by red staining) revealing no staining in retinal blood vessels (arrow) of a normal aged human specimen (A), while intense staining in blood vessels (arrow) of retinal lesions of human neovascular retinopathy specimens (PDR) (B,C). Images are oriented with the vitreous cavity (v) of the eye at the bottom of the panels although the fibrovascular lesion in the neovascular retinopathy image in C takes up the whole panel. (D-F) Immunohistochemical analysis of the distribution Albumin (shown by brown staining) of the specimens respectively shown in A through C revealing some staining in retinal blood vessels (arrow) but not in the neural retina of a normal aged human specimen (D), while intense staining in blood vessels (arrow) and neural retina of fibrovascular lesions of human neovascular retinopathy specimens (PDR) (E,F). A-F: 200×, scale bar=100 µm, representative images shown. (G) Levels of activated SFK in retinal blood vessels are expressed as fold of staining over a reference normal aged specimen. Values for age-matched normal are significantly different than the neovascular values (*P=*0.000007). Data is expressed as mean±s.e.m. of 3 duplicate experiments in PDR group and 6 duplicates in the aged normal group.

Since previous studies have shown that Tbdn expression is suppressed in retinal lesions of patients with PDR (Gendron et al., 2001) and that Albumin hyperpermeability is a feature of these lesions (Vinores et al., 1989, 1990, 1993; Knudsen et al., 2002), we next investigated if the levels of expression of activated SFK were altered in human eye specimens procured from donors with PDR. Immunohistological analyses with phospho-Src family (Tyr416) antibody revealed increased levels of activated SFK in the blood vessels of retinal lesions of human neovascular retinopathy specimens compared to normal human specimens (Fig. 8A-C). To confirm that retinal lesions of neovascular retinopathy specimens exhibit Albumin hyperpermeability as previously reported (Vinores et al., 1989, 1990, 1993; Knudsen et al., 2002), Albumin distribution of expression was analyzed on the same specimens. Expression of Albumin in control normal specimens was restricted to the retinal blood vessels while minimal or no levels of Albumin were found in the neural retina (Fig. 8D). In contrast, the retinal lesions of neovascular retinopathy specimens with increased levels of activated SFK displayed extravasation of Albumin from retinal blood vessels into the neural retina (Fig. 8E,F). Further quantitative analysis showed that neovascular retinopathy specimens had a significant increase (8-fold) in the levels of activated SFK in blood vessels of retinal lesions compared to normal specimens (*P*=0.000007; Fig. 8G).

## DISCUSSION

Previous studies have shown that Tbdn is an important regulator of endothelial permeability to Albumin in the retina (Paradis et al., 2008; Gendron et al., 2010). Both *in vitro* and *in vivo* experiments have revealed that the knockdown of Tbdn in retinal endothelial cells (Wall et al., 2004) leads to increases in Albumin permeability (Paradis et al., 2008; Gendron et al., 2010). In this study, we investigated the effects of Tbdn on components of the Albumin permeability signaling pathway and have explored the role of Tbdn as a modifier of permeability in retinal blood vessels *in vitro* and *in vivo*. Here we provide new evidence that Tbdn function in retinal endothelial cells involves the regulation of SFK and the c-Src substrate Cortactin. Tbdn knockdown in retinal endothelial cells resulted in up-regulation of the levels of activated SFK (c-Src, Fyn and Lyn) and phospho-Cortactin (Tyr421) (Figs 1-3). SFK members including c-Src, Blk, Fgr, Fyn, Hck, Lck, Lyn, Yes and Yrk, are known to mediate a wide variety of cellular processes (Kim et al., 2009). Among them, c-Src is the most extensively studied and plays a central role in endothelial cells in the regulation of transcellular permeability to Albumin (Kim et al., 2009), a process which involves endocytosis. Similarly, a role for Fyn but not Lyn has been described for increasing transcellular permeability of microvascular endothelial cells to Albumin (Mehta and Malik, 2006; Gong et al., 2008).

A role for Cortactin activation by c-Src in endocytic pathways in endothelial cells has previously been reported (Ammer and Weed, 2008). The present study revealed a direct role for Cortactin as a c-Src substrate in transendothelial Albumin permeability. There is evidence that Cortactin is also a target of Fyn (Ammer and Weed, 2008) but whether or not Lyn participates in Cortactin phosphorylation is not yet clear. We observed differences in expression patterns of SFK members Fyn and Lyn in RF/6A retinal endothelial cells versus mouse retinal tissues, with c-Src predominating in retinal tissues. Moreover, only limited activation of Fyn was observed in RF/6A while Gong et al. (2008) have presented evidence that Lyn is not involved in the regulation of microvascular endothelial permeability to Albumin (Gong et al., 2008). Taken together with our data, this suggests that it is c-Src activation alone that ultimately might be most important for Tbdn loss related retinal hypermeability to Albumin.

Previous studies have shown that Tbdn is found in a complex with Cortactin and negatively regulates Albumin permeability (Paradis et al., 2008). Our present results indicate that Tbdn expression negatively regulates the levels of activation of SFK and phosphorylation of Cortactin on Tyr421 in retinal endothelial cells. Our results also show that Tbdn knockdown retinal endothelial cells maintain a stimulatory response to Albumin reflected by a transient up-regulation of the levels of activated SFK (c-Src, Fyn and Lyn). This indicates that when Tbdn levels are reduced in these cells, the transcellular pathway is still responsive to Albumin while the basal levels of activation of SFK are higher than normal. These results suggest that Tbdn is involved in the regulation of basal levels of activation of SFK. Our results lead to the question of how SFK might be affected by Tbdn in endothelial cells. To date several mechanisms that regulate SFK activity have been elucidated (reviewed in Ingley, 2008). SFK are negatively regulated by intramolecular interactions which maintain the holoenzyme in a closed conformation. These interactions are mediated between the SH2 domain and a C-terminal tyrosine phosphorylated (Tyr527 in c-Src) motif, and between the SH3 domain and an internal proline-rich domain. The C-terminal Src kinase, Csk, or the Csk-homologous kinase, Chk, mediates the phosphorylation of Tyr527 necessary for the intramolecular interaction with the SH2 domain. Competitive binding of other proteins containing proline-rich domains and phospho-tyrosine containing motifs to respectively the SH3 and SH2 domains of c-Src allows a switch to an opened conformation. Complete stable activation of c-Src occurs through transmolecular autophosphorylation of Tyr416. Once activated, c-Src phosphorylates its substrates. Activated c-Src also interacts with Csk binding protein (Cbp), a transmembrane adaptor protein linked to the actin cytoskeleton. Cbp recruits Csk to the complex, allowing phosphorylation of c-Src at Tyr527 (Ingley, 2008; Hu et al., 2008). There is evidence that the critical step in the inactivation of c-Src is mediated by dephosphorylation of Tyr416 by Proline-Enriched Protein Phosphatase (PEP) which interacts with the SH3 domain of Csk. The above regulatory processes of SFK activity likely underlie the transient increases in the levels of activated SFK we observed upon Albumin stimulation of retinal endothelial cells (Fig. 1). Whether Tbdn is involved either directly or indirectly in these processes is not known and will require further study. Nevertheless, the cellular activities that inactivate SFK once activated shortly after stimulation of retinal endothelial cells with Albumin appears to be as effective in Tbdn knockdown cells as they are in control cells (Fig. 1).

It is not known if the regulation of the basal levels of activity of SFK differs from the regulatory processes controlling SFK activated by extracellular stimuli such as Albumin or growth factors. Mechanisms known to activate c-Src also include interference with the negative regulation of intramolecular binding (e.g. SH3 and proline-rich polylinker interaction or SH2 and C-terminal phospho-tyrosine motif containing Tyr527 interaction) (Engen et al., 2008). Binding of SFK to substrates or other adaptor molecules via their SH3 or SH2 domains is sufficient to induce kinase activation (Engen et al., 2008). Cortactin contains a consensus c-Src SH2 binding sequence which is adjacent to Tyr421 that was shown to bind the SH2 domain of c-Src. It is thought that this interaction maintains a state of c-Src which is phosphorylated at Tyr416, thereby stabilizing activated c-Src and its ability to phosphorylate Cortactin (Weed and Parsons, 2001). Since Cortactin is relatively more abundant than c-Src (20 times more whole cell lysate is required to detect c-Src compared to Cortactin), we hypothesized that the increase in the levels of phospho-Cortactin (Tyr421) we observed in Tbdn knockdown cells may be sufficient to stabilize activated c-Src and increase the basal levels of activity of c-Src. Our results revealed that Cortactin plays a role in regulating the levels of activated SFK in Tbdn knockdown retinal endothelial cells. The knockdown of Cortactin expression by siRNA led to a decrease in the levels of activated c-Src-Fyn as well as a decrease in Albumin permeability. These results indicate that Cortactin regulates Albumin permeability and Src family of kinases activation in retinal endothelial cells knocked down for Tbdn. These results are consistent with the hypothesis that the increase in the levels of phospho-Cortactin (Tyr421) by Tbdn suppression mediates the increase in activated SFK and permeability in retinal endothelial cells observed. The exact mechanism by which Tbdn regulates Cortactin phosphorylation on Tyr421 will require further study.

To examine if SFK activity up-regulation had a role in Tbdn knockdown-mediated increase in permeability to Albumin, we used the SFK inhibitor SKI-606 or *c-Src* siRNA. SKI-606 treatments and transfection of *c-Src* siRNA of retinal endothelial cells knocked down for Tbdn were effective, respectively, to reduce the levels of activated SFK and the levels of c-Src. Moreover, both treatments resulted in a decrease in the levels of phospho-Cortactin (Tyr421). Inhibition of SFK using low doses of SKI-606 reduced Albumin permeability of Tbdn knockdown retinal endothelial cells to levels observed in cells expressing normal levels of Tbdn (Fig. 5). However, treatment with either high concentrations of SKI-606 (10 µM and above) or *c-Src* siRNA yielding to significant reduction in the levels of activated SFK rendered the retinal endothelial cell monolayers leaky. As previously reported in other cell types (Golas et al., 2003; Coluccia et al., 2006; Zheng et al., 2008; Elliott et al., 2011), treatments of retinal endothelial cells with high concentrations of SKI-606 or c-Src knockdown were also associated with reduced cell survival which likely accounts for the leakiness we observed. For the Albumin transcytosis assay that is used in the present study it is critical that the cell monolayer has no spaces between the cells. A reduction in either survival or adhesion of the cells to the substrata or to each other would create open spaces between the cells allowing Albumin to cross the cell monolayer by a mechanism that is independent to transcytosis. As a result, the effect of either SKI-606 treatment or *c-Src* siRNA transfection on the Albumin permeability would be dependent on cell survival. As the concentrations of SKI-606 or the amount of *c-Src* siRNA were increased, larger amounts of cell death were observed and likely were associated with increasingly more interference with the Albumin transcytosis assay. c-Src plays numerous roles in various cellular processes that are critical to endothelial cell function including proliferation, survival, permeability, and adhesion (Kim et al., 2009). Therefore, our results suggest that Cortactin may be a better suited target to regulate retinal endothelial permeability to Albumin as its interference did not lead to a reduction in cell survival.

PDR is characterized by retinal neovascularization and retinal blood vessel hyperpermeability (Knudsen et al., 2002; Antonetti et al., 2012). Previous studies have shown that Tbdn expression is downregulated in the retinal blood vessels of neovascular lesions of PDR specimens (Gendron et al., 2001) as well as other neovascular retinopathies (Gendron et al., 2006, 2010). Conditional knockdown of Tbdn expression in mouse endothelium results in a pathological phenotype which is similar to the pathologies observed in human neovascular retinopathy (Wall et al., 2004; Gendron et al., 2006, 2010; Paradis et al., 2008). Our present data in mouse and human specimens provide further evidence that the loss of Tbdn expression in retinal blood vessels is a contributing factor that predisposes the retina to the development of pathology associated with hyperpermeability to Albumin and neovascularization involving c-Src activation. In the present study, analyses of a conditional endothelial Tbdn knockdown mouse model confirmed the up-regulation of activated c-Src in blood vessels of retinal lesions reaching levels up to 10-fold above control. Moreover, our analyses herein also revealed a significant increase in the levels of activated SFK in the retinal blood vessels in the ocular lesions of patients with PDR displaying hyperpermeability to Albumin. Deregulation of the activation of c-Src has been associated with vascular hyperpermeability in a range of studies (reviewed in Mehta and Malik, 2006). Increased endothelial permeability usually causes abnormal extravasation of blood components such as Albumin and accumulation of fluid in the extravascular space (Hu et al., 2008). Furthermore, these processes have been associated with inflammation and the recruitment of cytokines and growth factors to their cognate receptors in tissues which can lead to the activation of SFK (Kim et al., 2009; Kumar et al., 2009). These processes may explain elevated staining levels of activated SFK in retinal lesions in the tissues surrounding the retinal vasculature as well as the blood vessels.

In light of past work from our laboratory on the role of Tbdn loss in endothelial hyperpermeability in retinopathy (Paradis et al., 2002, 2008; Wall et al., 2004; Gendron et al., 2006, 2010), the work herein provides evidence that Tbdn acts through the c-Src/Cortactin pathway to maintain homeostasis of retinal blood vessels. Deregulation of c-Src has been linked with a broad spectrum of tissue changes in diseases including loss of growth control in cancers and barrier dysfunction in vasculopathy (Kim et al., 2009). Our present studies add to this knowledge in providing evidence that Tbdn regulation of retinal endothelial permeability involves c-Src activation and Cortactin phosphorylation (Tyr421).

## MATERIAL AND METHODS

### Cell culture

RF/6A, rhesus macaque choroid-retina (American Type Culture Collection, Manassas, VA, USA) and IEM, mouse embryonic endothelial cell lines were grown as previously described (Gendron et al., 1996, 2001). RF/6A clones knocked down for Tbdn expression by stable transfection of an antisense *Tbdn* cDNA construct *ASTbdn* and negative control clones have been described previously (Paradis et al., 2002, 2008). To monitor the activation of the Albumin permeability pathway, RF/6A cells were plated at 1.8×10^4^ cells/cm^2^ in growth media for 4 to 16 h. Fetal bovine serum (FBS) concentration was next reduced to 0.5% for 48 h followed by 3 h culture in serum-free media. Cell monolayers were then treated for 5 or 10 min with 5 µg/ml of bovine serum Albumin (BSA) (ICN Biomedicals Inc., Aurora, OH, USA).

For SKI-606 (Bosutinib; Biovision, Mountain View, CA, USA) treatment***,*** cells were plated at either 1.8×10^4^ or 9.1×10^4^ cells/cm^2^. The cells were pre-treated with various concentrations of SKI-606 or vehicle alone for either 3 h or 20 h.

For transcellular Albumin permeability assays, RF/6A cells and stable AS*Tbdn* and control clones were seeded at 9.1×10^4^ cells/cm^2^ onto 1%-gelatin-coated polystyrene filter inserts (Costar Transwell, no. 3470, 6.5-mm diameter, 0.33 cm^2^, 0.4-μm pore size; Corning, Tewksbury, MA, USA) 24 h prior to the assay. Three hours prior to the assay, the growth media were substituted to serum-free media.

### Mouse specimens

Endothelial Tbdn expression was knocked down in *TIE2/rtTA/Enh-TRE*/*ASTBDN* bi-transgenic middle aged mice using dietary Doxycycline (Dox Diet; 600 mg/kg; Bio-Serv, Frenchtown, NJ, USA), as previously described (Wall et al., 2004). Controls included age-matched mice fed a regular diet and age-matched single transgenic mice (*TIE2/rtTA/Enh* mice or *TRE*/*ASTBDN* mice) fed with Dox diet for the same length of time. Mice were sacrificed after either 1, 2, or 6 weeks of administration of Dox diet. Eye sections were analyzed histologically as described previously (Wall et al., 2004; Gendron et al., 2010) to map the progression of choroid-retinal pathology. Mouse retinae were surgically dissected from the sclera, vitreous and other ocular tissues under a dissecting microscope and whole cell lysates (WCL) were prepared for western blots. The care and use of animals in this study followed the guidelines set by the Canadian Council on Animal Care and were approved by the Institutional Animal Care Committee of Memorial University of Newfoundland.

### Human eye specimens

Human eye specimens were obtained from The National Disease Research Interchange (Philadelphia, PA, USA) or from the University of San Francisco Department of Ophthalmologic Pathology. Normal specimens (*n*=6) were from donors with an age range of 71 to 91 years old. The PDR specimens (*n*=3), some with retinal detachment and developed pre-retinal membrane, were from patients with age range of 60 to 74 years old. All PDR specimens have been previously reported to show suppression of Tbdn expression in the blood vessels of retinal lesions (Gendron et al., 2001). All research on human specimens followed the tenets of the Declaration of Helsinki and was performed under approval from the Health Research Ethics Board of Newfoundland and Labrador.

### Antibodies

Purified rabbit anti-Tbdn C755-766 antibody and purified mouse monoclonal anti-Tbdn antibody (clone OE5) were derived as described previously (Martin et al., 2007; Paradis et al., 2008; Gendron et al., 2010). Other antibodies used in this study include mouse monoclonal Cortactin 4F11 (Millipore, Billerica, MA, USA), mouse monoclonal c-Src clone 327 (Abcam, Cambridge, MA, USA) and mouse monoclonal α-Tubulin antibody (DM1A; Sigma, St. Louis, MO, USA). Rabbit polyclonal phospho-Cortactin (Tyr421) and phospho-Src family (Tyr416) antibodies were purchased from Cell Signaling Technology (Danvers, MA, USA) and goat anti-Albumin horseradish peroxidase (HRP)-conjugated antibody from GeneTex (San Antonio, TX, USA). Mouse monoclonal Fyn (sc-434) and Lyn (sc-7274) antibodies, and rabbit polyclonal ERK1 (sc-94) and Stat3 (sc-482) antibodies were obtained from Santa Cruz Biotechnology (Santa Cruz, CA, USA). Negative control mouse IgG_1_ or IgG_2a_ antibodies were obtained from Dako Canada (Mississauga, ON, Canada). Affinity purified horseradish peroxidase (HRP) conjugated-anti-rabbit IgG and -anti-mouse IgG reagents (Promega, Madison, WI, USA) were also used as secondary antibodies for western blot analyses. Alkaline phosphatase (AP) conjugated-anti-mouse IgG and -anti-rabbit IgG were obtained from Promega, while -anti-mouse IgG_2a_ was obtained from Vector Laboratories, Inc. (Burlingame, CA, USA) and were used for immunohistochemistry.

### siRNA transfections

All siRNA duplexes were purchased from Dharmacon (Thermo Fischer, Lafayette, CO, USA) with 3′ UU overhangs and were verified for their target specificity. SiRNA targeting *Macaca mulatta Tbdn* (5′-UGCGAGAUCUUGAGGGUUA-3′) and a matching scrambled non-silencing control siRNA (5′-GAUCCGUUCAUCGUCACUA-3′) were used at 10 nM and 20 nM. SiRNA duplex targeting *Macaca mulatta Cortactin* (5′-AUGCAACUUAUUGUAUCUGAA-3′) previously shown to knockdown Cortactin in other cells (Schnoor et al., 2011) and a matching scrambled non-silencing control siRNA (5′-AUGUACACGUAUUAUCGUAUA-3′) were used at 10 nM and 20 nM. SiRNA duplex targeting *Macaca mulatta c-Src* (5′-GAAGCUGAGGCAUGAGAAG) previously shown to knockdown c-Src in other cells (Zheng et al., 2008) and a matching scrambled non-silencing control siRNA (5′-GCUAAGAGAGUACGGAGAG-3′) were used at 1 nM, 2.5 nM, 5 nM, 10 nM and 50 nM. RF/6A cells were electroporated with siRNA using the Neon Transfection system (Invitrogen, Carlsbad, CA, USA) following manufacturer's protocols. The pmaxGFP vector (Lonza, Cologne, Germany) was used to monitor transfection efficiency which ranged from experiment to experiment from ∼70 to ∼80%. Electroporated RF/6A cells were cultured for 72 h before harvesting for western blot analysis or cultured for 48 h and re-plated for testing permeability and monitoring protein levels 24 h later.

### Western blot analysis and immunoprecipitation

WCL for western blot analyses were prepared in Triton lysis buffer (50 mM Tris-HCl pH 7.6, 150 mM NaCl, 0.5% [w/v] sodium deoxycholate, 0.1% [w/v] SDS, 1% [v/v] Triton X-100 and 10% [v/v] glycerol) supplemented with 1 mM dithiothreitol, 10 µg/ml leupeptin, 0.3 U/ml aprotinin, 1 mM phenylmethylsulfonyl fluoride (PMSF), 1 mM sodium orthovanadate, 50 mM sodium fluoride and 25 mM β-glycerophosphate as previously described (Paradis et al., 2008). Lysates were clarified by centrifugation and protein concentration determined by Bio-Rad Protein Assay kit (Bio-Rad Laboratories, Hercules, CA) using bovine serum Albumin (BSA) as the standard. For western blots of WCL equal quantities of protein from each sample were loaded onto an SDS-PAGE and transferred to nitrocellulose membrane (Bio-Rad Laboratories).

For immunoprecipitations, WCL were prepared in 50 mM Tris-HCl pH 7.6, 150 mM NaCl and 0.5% Brij 96 supplemented with protease and phosphatase inhibitors as above. Lysates were incubated with specific antibody or isotype-matched negative control mouse IgG_1_ or IgG_2a_ or no antibody control. Immune complexes were purified with protein-G–Sepharose beads (GE Healthcare, Buckinghamshire, UK). Immunoprecipitates were analyzed by SDS-PAGE and western blot.

For western blots, membranes were incubated with specific antibodies in 2% ECL Prime Blocking Reagent (GE Healthcare) in 10 mM Tris-HCl pH 7.6 and 150 mM NaCl (TBS) with 0.05% Tween 20 (TBST) with the exception of phosphorylated epitopes in which incubations with primary antibodies were performed in 5% BSA (MP Biomedicals, LLC., Solon, OH, USA) in TBST. Chemiluminescence detection reagents (GE Healthcare; KPL, Gaithersburg, MD, USA) and a Kodak Gel Logic 2200 imaging system (Eastman Kodak Company, Rochester, NY, USA) with Carestream Molecular imaging software (Woodbridge, CT, USA) were used for revealing protein expression signals and quantify protein levels by densitometry.

### Transcellular permeability assay

Albumin transcellular permeability assays were performed and analyzed following previously published methodology in serum free-media (Paradis et al., 2008). The transit of FITC-Albumin (Sigma) across confluent cell monolayers were monitored in triplicates at 0, 20 and 40 min.

### Immunohistochemistry

Immunohistochemistry was performed on paraffin-embedded sections of eye specimens processed as previously described (Paradis et al., 2002; Gendron et al., 2010). Sections (5 µm) were deparaffinized, post-fixed and washed in TBS. Samples were then blocked in 2% ECL Prime Blocking Agent in appropriate buffer (TBS or TBST) for 1 h. Incubations with antibodies were performed in blocking agent in buffer with the exception of phospho-Src family (Tyr416) antibody for which the incubation was performed in 5% BSA in TBS. For Tbdn and phospho-Src family (Tyr416) staining, sections were developed using appropriate secondary AP-conjugated antibody and Vector Red AP substrate with Levamisole (Vector Laboratories Inc.). For Albumin staining, Melanin was bleached by incubating the sections with 0.25% KMnO_4_ followed by 1% oxalic acid. The tissues endogenous peroxidase activity was blocked by treating the sections with 0.3% H_2_O_2_. Sections were stained with goat anti-Albumin-HRP-conjugated antibody while negative control sections were stained with HRP-conjugated goat anti-rabbit IgG at the same concentration as the anti-Albumin antibody. Sections were developed with NovaRED HRP substrate kit (Vector Laboratories Inc.). Sections were air dried and mounted with Permount (Fisher Scientific, Pittsburg, PA).

**S**ections were photographed for quantitation of staining as previously described (Gendron et al., 2010). Tbdn and phospho-Src family (Tyr416) levels in blood vessels of retinal lesions or controls were expressed as the average staining levels of at least three separate specimens. Intensity of staining in retinal blood vessels and background staining (from retinal blood vessels stained with negative control antibody or from non vascular areas such as red blood cells or photoreceptors) were measured by determining the ratio of red color/green color intensity using HIS Colourspy tool of Openlab software as previously described (Martin et al., 2007; Gendron et al., 2006). Negative control antibody produced minimal background. Levels of staining were calculated by subtracting the background measurements. Relative intensities were expressed as the average staining levels±standard error (s.e.m.).

### Statistical analyses

All quantitative analyses were compared using the two-tailed Student's *t*-test with Microsoft Excel or ANOVA with Prism. The data was considered to be statistically significant if the *P* value was less than or equal to 0.05.

## Supplementary Material

Supplementary Material

## References

[BIO010496C1] Ammer A. G. and Weed S. A. (2008). Cortactin branches out: roles in regulating protrusive actin dynamics. *Cell Motil. Cytoskeleton* 65, 687-707. 10.1002/cm.20296 18615630 PMC2561250

[BIO010496C2] Antonetti D. A., Klein R. and Gardner T. W. (2012). Diabetic retinopathy. *N. Engl. J. Med.* 366, 1227-1239. 10.1056/NEJMra1005073 22455417

[BIO010496C3] Asaumi M., Iijima K., Sumioka A., Iijima-Ando K., Kirino Y., Nakaya T. and Suzuki T. (2005). Interaction of N-terminal acetyltransferase with the cytoplasmic domain of beta-amyloid precursor protein and its effect on A beta secretion. *J. Biochem.* 137, 147-155. 10.1093/jb/mvi014 15749829

[BIO010496C4] Bhutto I. and Lutty G. (2012). Understanding age-related macular degeneration (AMD): relationships between the photoreceptor/retinal pigment epithelium/Bruch's membrane/choriocapillaris complex. *Mol. Aspects Med.* 33, 295-317. 10.1016/j.mam.2012.04.005 22542780 PMC3392421

[BIO010496C5] Campochiaro P. A. and Hackett S. F. (2003). Ocular neovascularization: a valuable model system. *Oncogene* 22, 6537-6548. 10.1038/sj.onc.1206773 14528278

[BIO010496C6] Cao H., Orth J. D., Chen J., Weller S. G., Heuser J. E. and McNiven M. A. (2003). Cortactin is a component of clathrin-coated pits and participates in receptor-mediated endocytosis. *Mol. Cell. Biol.* 23, 2162-2170. 10.1128/MCB.23.6.2162-2170.2003 12612086 PMC149460

[BIO010496C7] Cao H., Chen J., Krueger E. W. and McNiven M. A. (2010). SRC-mediated phosphorylation of dynamin and cortactin regulates the “constitutive” endocytosis of transferrin. *Mol. Cell. Biol.* 30, 781-792. 10.1128/MCB.00330-09 19995918 PMC2812239

[BIO010496C8] Coluccia A. M. L., Benati D., Dekhil H., De Filippo A., Lan C. and Gambacorti-Passerini C. (2006). SKI-606 decreases growth and motility of colorectal cancer cells by preventing pp60(c-Src)-dependent tyrosine phosphorylation of beta-catenin and its nuclear signaling. *Cancer Res.* 66, 2279-2286. 10.1158/0008-5472.CAN-05-2057 16489032

[BIO010496C9] Cosen-Binker L. I. and Kapus A. (2006). Cortactin: the gray eminence of the cytoskeleton. *Physiology* 21, 352-361. 10.1152/physiol.00012.2006 16990456

[BIO010496C10] Daly R. J. (2004). Cortactin signalling and dynamic actin networks. *Biochem. J.* 382, 13-25. 10.1042/BJ20040737 15186216 PMC1133910

[BIO010496C11] Elliott J., Zheleznova N. N. and Wilson P. D. (2011). c-Src inactivation reduces renal epithelial cell-matrix adhesion, proliferation, and cyst formation. *Am. J. Physiol. Cell Physiol.* 301, C522-C529. 10.1152/ajpcell.00163.2010 21508333 PMC3154563

[BIO010496C12] Engen J. R., Wales T. E., Hochrein J. M., Meyn M. A. III, Banu Ozkan S., Bahar I. and Smithgall T. E. (2008). Structure and dynamic regulation of Src-family kinases. *Cell. Mol. Life Sci.* 65, 3058-3073. 10.1007/s00018-008-8122-2 18563293 PMC9357288

[BIO010496C13] Friedlander M., Theesfeld C. L., Sugita M., Fruttiger M., Thomas M. A., Chang S. and Cheresh D. A. (1996). Involvement of integrins alpha v beta 3 and alpha v beta 5 in ocular neovascular diseases. *Proc. Natl. Acad. Sci. USA* 93, 9764-9769. 10.1073/pnas.93.18.9764 8790405 PMC38503

[BIO010496C14] Geissenhoner A., Weise C. and Ehrenhofer-Murray A. E. (2004). Dependence of ORC silencing function on NatA-mediated Nalpha acetylation in Saccharomyces cerevisiae. *Mol. Cell. Biol.* 24, 10300-10312. 10.1128/MCB.24.23.10300-10312.2004 15542839 PMC529049

[BIO010496C15] Gendron R. L., Tsai F.-Y., Paradis H. and Arceci R. J. (1996). Induction of embryonic vasculogenesis by bFGF and LIF in vitro and in vivo. *Dev. Biol.* 177, 332-346. 10.1006/dbio.1996.0167 8660899

[BIO010496C16] Gendron R. L., Adams L. C. and Paradis H. (2000). Tubedown-1, a novel acetyltransferase associated with blood vessel development. *Dev. Dyn.* 218, 300-315. 10.1002/(SICI)1097-0177(200006)218:2<300::AID-DVDY5>3.0.CO;2-K 10842358

[BIO010496C17] Gendron R. L., Good W. V., Adams L. C. and Paradis H. (2001). Suppressed expression of tubedown-1 in retinal neovascularization of proliferative diabetic retinopathy. *Invest. Ophthalmol. Vis. Sci.* 42, 3000-3007. 11687548

[BIO010496C18] Gendron R. L., Good W. V., Miskiewicz E., Tucker S., Phelps D. L. and Paradis H. (2006). Tubedown-1 (tbdn-1) suppression in oxygen-induced retinopathy and in retinopathy of prematurity. *Mol. Vis.* 12, 108-116. 16518308

[BIO010496C19] Gendron R. L., Laver N. V., Good W. V., Grossniklaus H. E., Miskiewicz E., Whelan M. A., Walker J. and Paradis H. (2010). Loss of tubedown expression as a contributing factor in the development of age-related retinopathy. *Invest. Ophthalmol. Vis. Sci.* 51, 5267-5277. 10.1167/iovs.09-4527 20463314

[BIO010496C20] Giani A., Luiselli C., Esmaili D. D., Salvetti P., Cigada M., Miller J. W. and Staurenghi G. (2011). Spectral-domain optical coherence tomography as an indicator of fluorescein angiography leakage from choroidal neovascularization. *Invest. Ophthalmol. Vis. Sci.* 52, 5579-5586. 10.1167/iovs.10-6617 21693602

[BIO010496C21] Golas J. M., Arndt K., Etienne C., Lucas J., Nardin D., Gibbons J., Frost P., Ye F., Boschelli D. H. and Boschelli F. (2003). SKI-606, a 4-anilino-3-quinolinecarbonitrile dual inhibitor of Src and Abl kinases, is a potent antiproliferative agent against chronic myelogenous leukemia cells in culture and causes regression of K562 xenografts in nude mice. *Cancer Res.* 63, 375-381. 12543790

[BIO010496C22] Gong P., Angelini D. J., Yang S., Xia G., Cross A. S., Mann D., Bannerman D. D., Vogel S. N. and Goldblum S. E. (2008). TLR4 signaling is coupled to SRC family kinase activation, tyrosine phosphorylation of zonula adherens proteins, and opening of the paracellular pathway in human lung microvascular endothelia. *J. Biol. Chem.* 283, 13437-13449. 10.1074/jbc.M707986200 18326860 PMC2442341

[BIO010496C23] Hu G. and Minshall R. D. (2009). Regulation of transendothelial permeability by src kinase. *Microvasc. Res.* 77, 21-25. 10.1016/j.mvr.2008.10.002 19027754

[BIO010496C24] Hu G., Place A. T. and Minshall R. D. (2008). Regulation of endothelial permeability by src kinase signaling: vascular leakage versus transcellular transport of drugs and macromolecules. *Chem. Biol. Interact.* 171, 177-189. 10.1016/j.cbi.2007.08.006 17897637 PMC3001132

[BIO010496C25] Ingley E. (2008). Src family kinases: regulation of their activities, levels and identification of new pathways. *Biochim. Biophys. Acta* 1784, 56-65. 10.1016/j.bbapap.2007.08.012 17905674

[BIO010496C26] Kalvik T. V. and Arnesen T. (2013). Protein N-terminal acetyltransferases in cancer. *Oncogene* 32, 269-276. 10.1038/onc.2012.82 22391571

[BIO010496C27] Kim M. P., Park S. I., Kopetz S. and Gallick G. E. (2009). Src family kinases as mediators of endothelial permeability: effects on inflammation and metastasis. *Cell Tissue Res.* 335, 249-259. 10.1007/s00441-008-0682-9 18815812 PMC3907084

[BIO010496C28] Kimura Y., Saeki Y., Yokosawa H., Polevoda B., Sherman F. and Hirano H. (2003). N-terminal modifications of the 19S regulatory particle subunits of the yeast proteasome. *Arch. Biochem. Biophys.* 409, 341-348. 10.1016/S0003-9861(02)00639-2 12504901

[BIO010496C29] Knudsen S. T., Bek T., Poulsen P. L., Hove M. N., Rehling M. and Mogensen C. E. (2002). Macular edema reflects generalized vascular hyperpermeability in type 2 diabetic patients with retinopathy. *Diabetes Care* 25, 2328-2334. 10.2337/diacare.25.12.2328 12453981

[BIO010496C30] Komarova Y. and Malik A. B. (2010). Regulation of endothelial permeability via paracellular and transcellular transport pathways. *Annu. Rev. Physiol.* 72, 463-493. 10.1146/annurev-physiol-021909-135833 20148685

[BIO010496C31] Kumar P., Shen Q., Pivetti C. D., Lee E. S., Wu M. H. and Yuan S. Y. (2009). Molecular mechanisms of endothelial hyperpermeability: implications in inflammation. *Expert Rev. Mol. Med.* 11, e19 10.1017/S1462399409001112 19563700 PMC2828491

[BIO010496C32] Lannutti B. J., Minear J., Blake N. and Drachman J. G. (2006). Increased megakaryocytopoiesis in Lyn-deficient mice. *Oncogene* 25, 3316-3324. 10.1038/sj.onc.1209351 16418722

[BIO010496C33] Leto G., Pricci F., Amadio L., Iacobini C., Cordone S., Diaz-Horta O., Romeo G., Barsotti P., Rotella C. M., di Mario U. et al. (2001). Increased retinal endothelial cell monolayer permeability induced by the diabetic milieu: role of advanced non-enzymatic glycation and polyol pathway activation. *Diabetes Metab. Res. Rev.* 17, 448-458. 10.1002/dmrr.227 11757081

[BIO010496C34] Liszczak G., Goldberg J. M., Foyn H., Petersson E. J., Arnesen T. and Marmorstein R. (2013). Molecular basis for N-terminal acetylation by the heterodimeric NatA complex. *Nat. Struct. Mol. Biol.* 20, 1098-1105. 10.1038/nsmb.2636 23912279 PMC3766382

[BIO010496C35] Ljubimov A. V., Burgeson R. E., Butkowski R. J., Couchman J. R., Zardi L., Ninomiya Y., Sado Y., Huang Z. S., Nesburn A. B. and Kenney M. C. (1996). Basement membrane abnormalities in human eyes with diabetic retinopathy. *J. Histochem. Cytochem.* 44, 1469-1479. 10.1177/44.12.8985139 8985139

[BIO010496C36] Lutty G. A. (2013). Effects of diabetes on the eye. *Invest Ophthalmol. Vis. Sci.* 54, ORSF81-ORSF87. 10.1167/iovs.13-12979 24335073 PMC3864380

[BIO010496C37] Martin D. T., Gendron R. L., Jarzembowski J. A., Perry A., Collins M. H., Pushpanathan C., Miskiewicz E., Castle V. P. and Paradis H. (2007). Tubedown expression correlates with the differentiation status and aggressiveness of neuroblastic tumors. *Clin. Cancer Res.* 13, 1480-1487. 10.1158/1078-0432.CCR-06-1716 17332292

[BIO010496C38] Mehta D. and Malik A. B. (2006). Signaling mechanisms regulating endothelial permeability. *Physiol. Rev.* 86, 279-367. 10.1152/physrev.00012.2005 16371600

[BIO010496C39] Myklebust L. M., Van Damme P., Støve S. I., Dörfel M. J., Abboud A., Kalvik T. V., Grauffel C., Jonckheere V., Wu Y., Swensen J. et al. (2015). Biochemical and cellular analysis of Ogden syndrome reveals downstream Nt-acetylation defects. *Hum. Mol. Genet.* 24, 1956-1976. 10.1093/hmg/ddu611 25489052 PMC4355026

[BIO010496C40] Nagy J. A., Dvorak A. M. and Dvorak H. F. (2012). Vascular hyperpermeability, angiogenesis, and stroma generation. *Cold Spring Harb. Perspect. Med.* 2, a006544 10.1101/cshperspect.a006544 22355795 PMC3281587

[BIO010496C42] Paques M., Massin P. and Gaudric A. (1997). Growth factors and diabetic retinopathy. *Diabetes Metab.* 23, 125-130. 9137900

[BIO010496C43] Paradis H., Liu C.-Y., Saika S., Azhar M., Doetschman T., Good W. V., Nayak R., Laver N., Kao C. W.-C., Kao W. W. et al. (2002). Tubedown-1 in remodeling of the developing vitreal vasculature in vivo and regulation of capillary outgrowth in vitro. *Dev. Biol.* 249, 140-155. 10.1006/dbio.2002.0757 12217325

[BIO010496C44] Paradis H., Islam T., Tucker S., Tao L., Koubi S. and Gendron R. L. (2008). Tubedown associates with cortactin and controls permeability of retinal endothelial cells to albumin. *J. Cell Sci.* 121, 1965-1972. 10.1242/jcs.028597 18495841

[BIO010496C45] Schnoor M., Lai F. P. L., Zarbock A., Klaver R., Polaschegg C., Schulte D., Weich H. A., Oelkers J. M., Rottner K. and Vestweber D. (2011). Cortactin deficiency is associated with reduced neutrophil recruitment but increased vascular permeability in vivo. *J. Exp. Med.* 208, 1721-1735. 10.1084/jem.20101920 21788407 PMC3149227

[BIO010496C46] Sugiura N., Adams S. M. and Corriveau R. A. (2003). An evolutionarily conserved N-terminal acetyltransferase complex associated with neuronal development. *J. Biol. Chem.* 278, 40113-40120. 10.1074/jbc.M301218200 12888564

[BIO010496C47] Vinores S. A., Gadegbeku C., Campochiaro P. A. and Green W. R. (1989). Immunohistochemical localization of blood-retinal barrier breakdown in human diabetics. *Am. J. Pathol.* 134, 231-235. 2916645 PMC1879597

[BIO010496C48] Vinores S. A., Campochiaro P. A., Lee A., McGehee R., Gadegbeku C. and Green W. R. (1990). Localization of blood-retinal barrier breakdown in human pathologic specimens by immunohistochemical staining for albumin. *Lab. Invest.* 62, 742-750. 2193194

[BIO010496C49] Vinores S. A., Van Niel E., Swerdloff J. L. and Campochiaro P. A. (1993). Electron microscopic immunocytochemical evidence for the mechanism of blood-retinal barrier breakdown in galactosemic rats and its association with aldose reductase expression and inhibition. *Exp. Eye Res.* 57, 723-735. 10.1006/exer.1993.1180 8150024

[BIO010496C50] Wall D. S., Gendron R. L., Good W. V., Miskiewicz E., Woodland M., Leblanc K. and Paradis H. (2004). Conditional knockdown of tubedown-1 in endothelial cells leads to neovascular retinopathy. *Invest. Ophthalmol. Vis. Sci.* 45, 3704-3712. 10.1167/iovs.03-1410 15452080

[BIO010496C51] Wang X., Connelly J. J., Wang C. L. and Sternglanz R. (2004). Importance of the Sir3 N-terminus and its acetylation for yeast transcriptional silencing. *Genetics* 168, 547-551. 10.1534/genetics.104.028803 15454564 PMC1448112

[BIO010496C52] Weed S. A. and Parsons J. T. (2001). Cortactin: coupling membrane dynamics to cortical actin assembly. *Oncogene* 20, 6418-6434. 10.1038/sj.onc.1204783 11607842

[BIO010496C53] Zheng X., Resnick R. J. and Shalloway D. (2008). Apoptosis of estrogen-receptor negative breast cancer and colon cancer cell lines by PTP alpha and Src RNAi. *Int. J. Cancer* 122, 1999-2007. 10.1002/ijc.23321 18183590 PMC4878911

[BIO010496C54] Zhu J., Yu D., Zeng X.-C., Zhou K. and Zhan X. (2007). Receptor-mediated endocytosis involves tyrosine phosphorylation of cortactin. *J. Biol. Chem.* 282, 16086-16094. 10.1074/jbc.M701997200 17420251

