# Supplementary material for: Tubedown regulation of retinal endothelial permeability signaling pathways
Source: Biol Open. 2015 Jul 3;4(8):970–9. doi: 10.1242/bio.010496 (PMC4542279; doi:10.1242/bio.010496)
Supplement: Supplementary Material [file supp_4_8_970__index.html]

Tubedown regulation of retinal endothelial permeability signaling pathways — Supplementary Material 

# Tubedown regulation of retinal endothelial permeability signaling pathways

## BIO010496 Supplementary Material

- Supplementary Material
