# Supplementary material for: Tubedown regulation of retinal endothelial permeability signaling pathways
Source: Biol Open. 2015 Jul 3;4(8):970–9. doi: 10.1242/bio.010496 (PMC4542279; doi:10.1242/bio.010496)
Supplement: Supplementary Material [file supp_bio.010496_BIO010496supp.pdf]

Supplementary Figures

Supplementary Figure 1

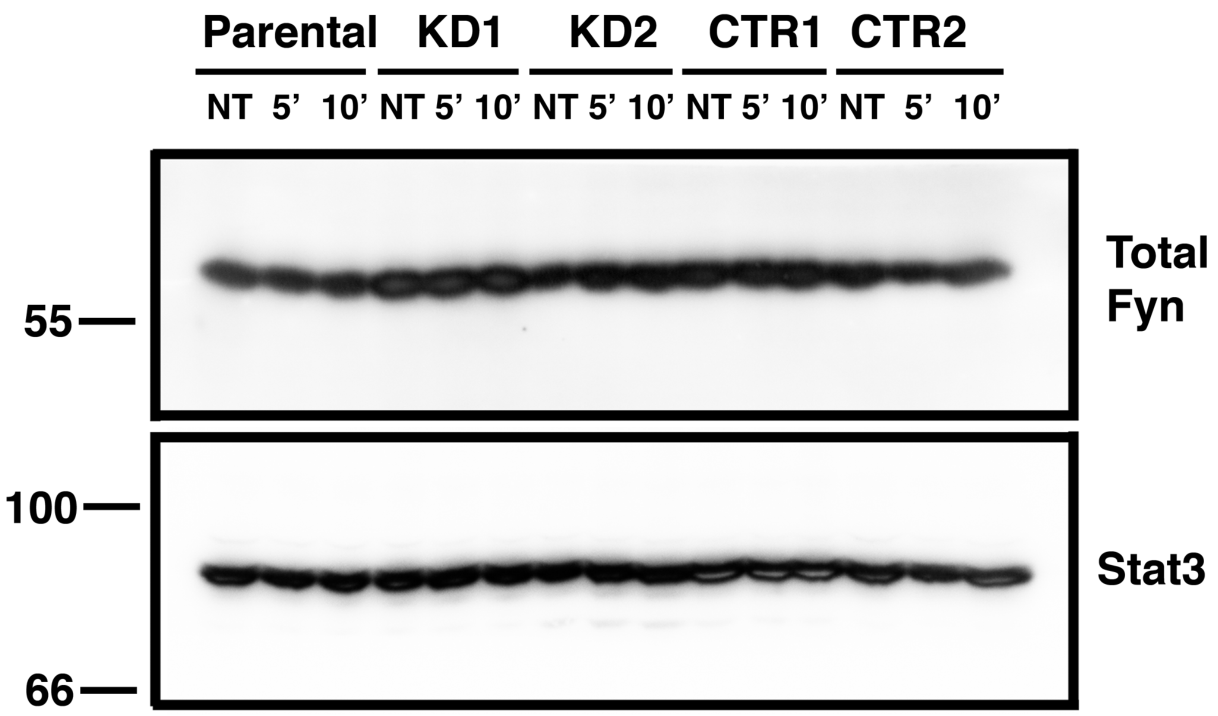

Analysis of Fyn expression levels in Tbdn knockdown retinal endothelial cell clones. RF/6A parental (Parental), Tbdn knockdown (KD 1, KD 2) and control (CTR 1, CTR 2) retinal endothelial cell clones were serum starved followed by no treatment (NT) or stimulation with serum Albumin for 5 (5') and 10 (10') minutes. Samples were analyzed by Western blotting for total Fyn levels and STAT3 for loading control and sample integrity. Quantitative analysis showed no significant changes in the levels of total Fyn over Stat3 among the different clones (not shown).

Supplementary Figure 2A:

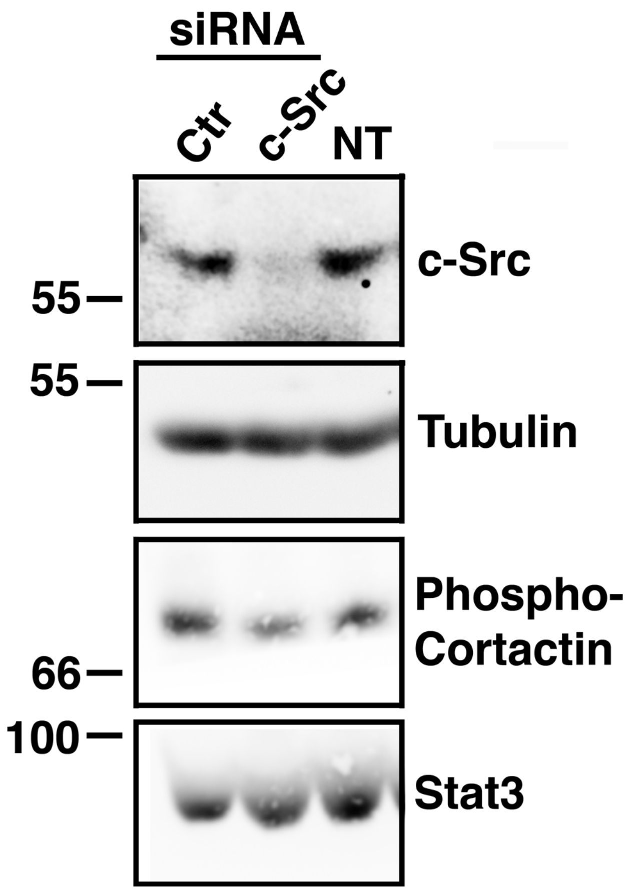

Supplementary Figure 2B

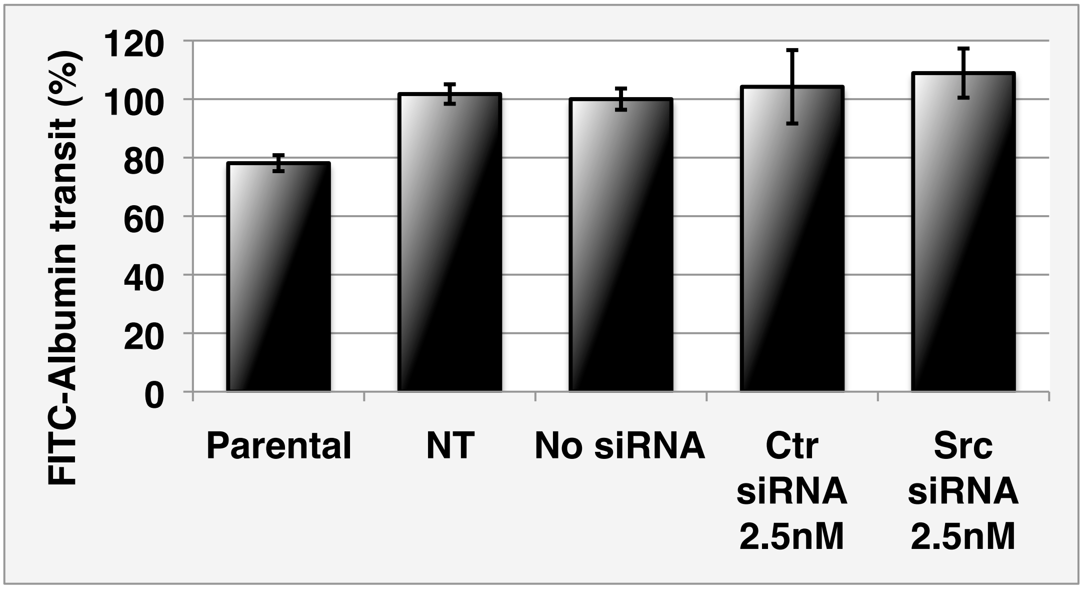

Supplementary Figure 2C

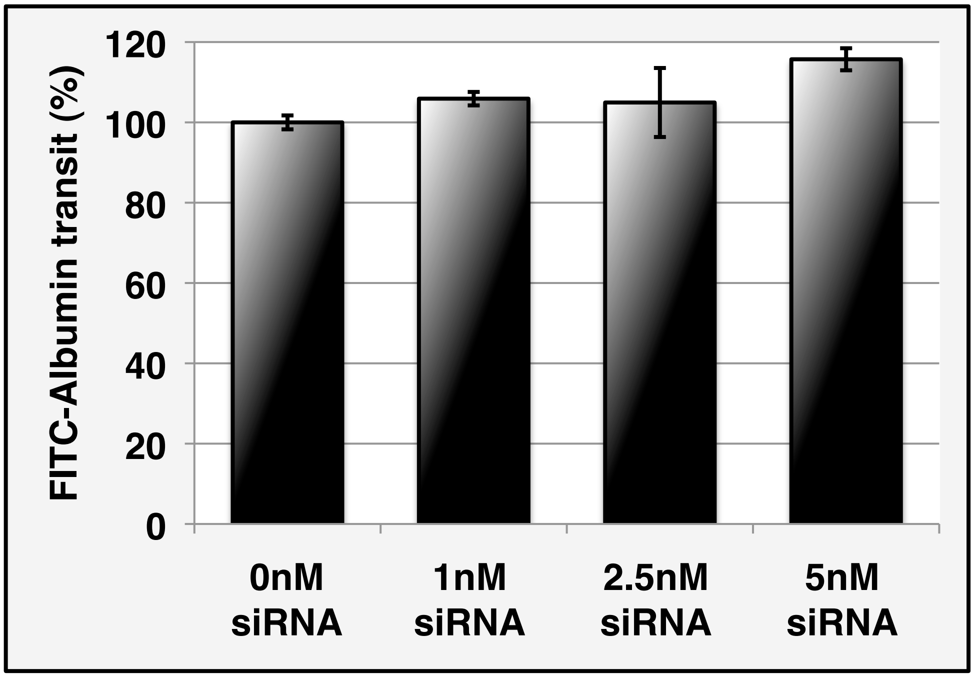

Effect of c-Src knockdown by siRNA on phospho-Cortactin (Tyr421) levels and Albumin permeability in retinal endothelial cells knocked down for Tbdn. C-Src expression was knocked down by transfection of *c-Src* siRNA (c-Src siRNA) compared to control siRNA (Ctr siRNA), no siRNA (NO siRNA). Non-transfected (NT) RF/6A retinal endothelial cells knockdown for Tbdn were also used as control. Samples were analyzed by Western blotting for levels of c-Src (c-Src), Tbdn (not shown), phospho-Cortactin (Tyr421), and Stat3 or Tubulin for loading control; and Albumin permeability assay. c-Src knockdown of ~40% or more by transfection of 2.5nM and above of c-Src siRNA resulted in a decrease in phospho-Cortactin (Tyr421) levels. (**A**) Representative Western blot analysis of cells transfected with 5nM of indicated siRNA and controls is shown. (**B)** The transit of FITC-Albumin across parental RF/6A cells compared to non-transfected (NT) or transfected (No siRNA, Ctr siRNA and Src siRNA) retinal endothelial cells knockdown for Tbdn was reduced ($P < 0.041$). Transfection of retinal

endothelial cells knockdown for Tbdn with 2.5nM of c-Src siRNA was not associated with a change in the transit of FITC-Albumin across the cell monolayer compared to controls (NT, No siRNA, and Ctr siRNA; $P \geq 0.33$). (**C)** Transfection of 5nM of c-Src siRNA increased ($P = 0.001$) the transit of FITC-Albumin across retinal endothelial cells knockdown for Tbdn while 1nM and 2.5nM of c-Src siRNA had no significant effect ($P \geq 0.09$) compared to control without siRNA (0nM siRNA). (**B and C)** Results are expressed as the means +/- S.E.M in percentage relative to control cells transfected without siRNA and represent the summary of 4 experiments (**B**) and 5 experiments (**C**).
